# Long-lasting blood pressure lowering effects of nitrite are NO-independent and mediated by hydrogen peroxide, persulfides, and oxidation of protein kinase G1α redox signalling

**DOI:** 10.1093/cvr/cvz202

**Published:** 2019-08-01

**Authors:** Martin Feelisch, Takaaki Akaike, Kayleigh Griffiths, Tomoaki Ida, Oleksandra Prysyazhna, Joanna J Goodwin, Nicholas D Gollop, Bernadette O Fernandez, Magdalena Minnion, Miriam M Cortese-Krott, Alessandra Borgognone, Rosie M Hayes, Philip Eaton, Michael P Frenneaux, Melanie Madhani

**Affiliations:** 1 Clinical and Experimental Sciences, Faculty of Medicine, University of Southampton, Southampton General Hospital, Southampton, SO16 6YD, UK; 2 Department of Environmental Medicine and Molecular Toxicology, Tohoku University Graduate School of Medicine, Sendai, 980-8575, Japan; 3 Institute of Cardiovascular Sciences, College of Medical and Dental Sciences, University of Birmingham, Birmingham, B15 2TT, UK; 4 King’s College of London, School of Cardiovascular Medicine & Sciences, The British Heart Foundation Centre of Excellence, The Rayne Institute, St Thomas' Hospital, London, SE1 7EH, UK; 5 Norwich Medical School, University of East Anglia, Bob Champion Research and Education Building, Norwich Research Park, Norwich, NR4 7UQ, UK; 6 Division of Cardiology, Pulmonology, and Vascular Medicine, Medical Faculty, Heinrich Heine University, Düsseldorf, 40225, Germany

**Keywords:** Nitrite, Blood pressure, Redox, Hydrogen peroxide, Persulfides

## Abstract

**Aims:**

Under hypoxic conditions, nitrite (${\text{NO}}_{2}^{\text{−}}$) can be reduced to nitric oxide (NO) eliciting vasorelaxation. However, nitrite also exerts vasorelaxant effects of potential therapeutic relevance under normal physiological conditions via undetermined mechanisms. We, therefore, sought to investigate the mechanism(s) by which nitrite regulates the vascular system in normoxia and, specifically, whether the biological effects are a result of NO generation (as in hypoxia) or mediated via alternative mechanisms involving classical downstream targets of NO [e.g. effects on protein kinase G1α (PKG1α)].

**Methods and results:**

*Ex vivo* myography revealed that, unlike in thoracic aorta (conduit vessels), the vasorelaxant effects of nitrite in mesenteric resistance vessels from wild-type (WT) mice were NO-independent. Oxidants such as H_2_O_2_ promote disulfide formation of PKG1α, resulting in NO- cyclic guanosine monophosphate (cGMP) independent kinase activation. To explore whether the microvascular effects of nitrite were associated with PKG1α oxidation, we used a Cys42Ser PKG1α knock-in (C42S PKG1α KI; ‘redox-dead’) mouse that cannot transduce oxidant signals. Resistance vessels from these C42S PKG1α KI mice were markedly less responsive to nitrite-induced vasodilation. Intraperitoneal (i.p.) bolus application of nitrite in conscious WT mice induced a rapid yet transient increase in plasma nitrite and cGMP concentrations followed by prolonged hypotensive effects, as assessed using *in vivo* telemetry. In the C42S PKG1α KI mice, the blood pressure lowering effects of nitrite were lower compared to WT. Increased H_2_O_2_ concentrations were detected in WT resistance vessel tissue challenged with nitrite. Consistent with this, increased cysteine and glutathione persulfide levels were detected in these vessels by mass spectrometry, matching the temporal profile of nitrite’s effects on H_2_O_2_ and blood pressure.

**Conclusion:**

Under physiological conditions, nitrite induces a delayed and long-lasting blood pressure lowering effect, which is NO-independent and occurs via a new redox mechanism involving H_2_O_2,_ persulfides, and PKG1α oxidation/activation. Targeting this novel pathway may provide new prospects for anti-hypertensive therapy.

## Graphical Abstract

**Graphical Abstract:**
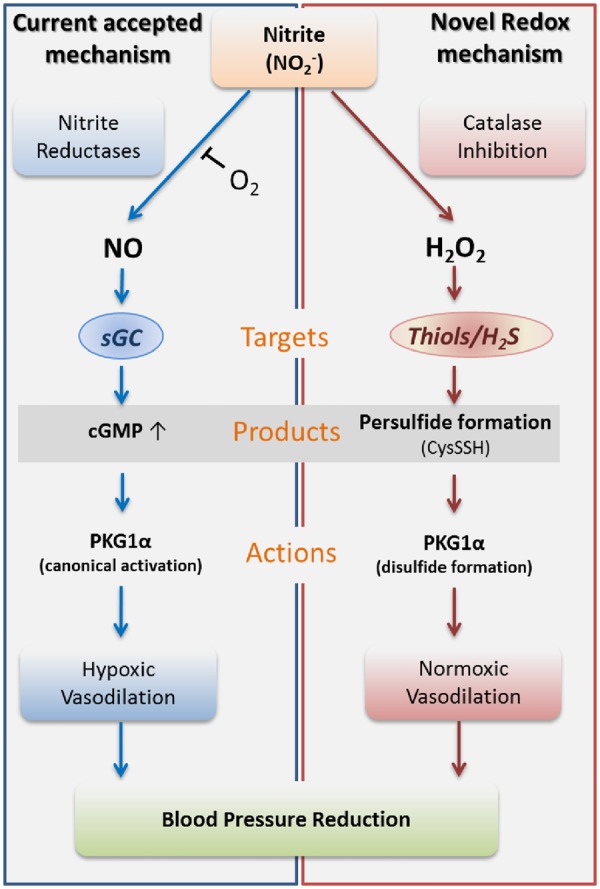

## 1. Introduction

Despite current pharmacotherapies and interventional procedures, arterial hypertension remains a global health burden and a major risk factor for cardiovascular diseases, dementia, renal failure, and premature death.^1^ Inorganic nitrite (NO_2_^−^) elicits vasorelaxation and lowers blood pressure when infused. Due to the potential beneficial effects and the possible therapeutic utility of NO_2_^−^ in treating hypertension, it becomes essential to understand the mechanism(s) and efficacy by which NO_2_^−^ elicits vasorelaxation under normoxia.^2^

Augmentation of plasma nitrite by oral administration of inorganic nitrate (converted to nitrite by oral/gut bacteria following enterosalivary circulation) has been shown to effectively reduce blood pressure in various animal models and in healthy human subjects.^2–5^ It is now clear that at least some of the pharmacological actions of nitrite are mediated via its reduction to nitric oxide (NO) and increased NO/cyclic guanosine monophosphate (cGMP)-signalling under conditions of low pH and oxygen tension.^6^^,^^7^ Whilst this nitrite-NO pathway offers a conceptually attractive explanation for the blood pressure lowering effect during hypoxia, nitrite also has vasorelaxant and blood pressure lowering effects under normoxic conditions. However, the mechanism(s) responsible for these effects remain incompletely understood. We have shown that under normoxic conditions, the rate of conversion of nitrite to NO is extremely slow,^8^ suggesting that these effects may be NO-independent. Using single intraperitoneal (i.p.) injections of nitrite in rats, we previously demonstrated that nitrite was rapidly absorbed and metabolized to nitrate and nitroso/nitrosyl products in all but two tissues studied (brain and aorta)^9^; this was accompanied by cardioprotection and a sustained (48 h) increase in ascorbate oxidation in the heart, reflecting persistent changes in tissue redox status.^10^

The mechanism responsible for the vasoactivity of NO (which can be generated from nitrite under hypoxic conditions) is well established and involves activation of the enzyme soluble guanylyl cylase (sGC), resulting in an increase in the generation of the second messenger cGMP with consecutive activation of the cGMP-binding kinase protein kinase G1α (PKG1α), which further transduces the signal associated with vasorelaxation.^11^ We have previously shown that PKG1α itself is subject to redox regulation such that the presence of oxidants can result in inter-protein disulfide formation, which translates into an increase in enzyme activity without changes in cGMP.^12^^,^^13^ Thus, species such as hydrogen peroxide (H_2_O_2_) that are capable of causing thiol to disulfide oxidation can elicit smooth muscle relaxation, and therefore, lead to vasodilation via oxidative activation of PKG1α in an NO-cGMP-independent fashion. Stubbert *et al.*^14^ also reported that polysulfur species derived from hydrogen sulfide (H_2_S) oxidation are capable of activating PKG1α via oxidation. The generation of polysulfur species and/or other sulfur oxidants (e.g. H_2_O_2_) therefore represents a NO-independent mechanism of PKG1α activation, which could also translate into vasorelaxation. Thus, if nitrite-mediated vasorelaxation under normoxic conditions was found to be NO-independent, it is possible that nitrite is capable of eliciting vasorelaxation via oxidative activation of PKG1α.

Recently, reactive sulfur species known as persulfides (RSSH) and polysulfides (RS_*n*_SR, RSS_*n*_H, *n* > 1) originating from sulfur-containing amino acid metabolism were detected in mammalian tissues and fluids.^15^ Specifically, cysteine persulfide (CysSSH), glutathione persulfide (GSSH) and related species may function as potent antioxidants, cytoprotectants, and redox signalling intermediates,^16^ and CysSSH appears to be involved in the regulation of mitochondrial biogenesis and bioenergetics.^17^ However, the physiological relevance and function of these species is incompletely understood, and whether nitrite has the ability to generate these polysulfur species is unknown.

The significance of nitrite as a redox regulator is not yet widely recognized, and evidence for the possible involvement of these pathways in its blood pressure lowering effects under physiological conditions is lacking. In the present study, we, therefore, sought to explore whether nitrite has the ability to mediate redox signalling and reduce vascular tone under normoxic conditions. We hypothesized that nitrite induces vasodilation and lowers blood pressure by generating endogenous thiol oxidants, such as H_2_O_2_ and persulfides, to eventually induce oxidation of PKG1α. We integrated *in vitro* and *in vivo* approaches to investigate the redox properties of nitrite. Our studies were further supported by the use of ‘redox-dead’ Cys42ser PKG1α knock-in (C42S PKG1α KI) mice, which are unable to transduce oxidant signals because the cysteinyl thiol has been replaced by a hydroxyl group, and therefore this mutation prevents the oxidative activation of PKG1α. We demonstrate that a single bolus application of nitrite induces long-lasting blood pressure lowering effects by generating H_2_O_2_ and persulfide species in the resistance vessels to activate PKG1α in an NO-independent manner.

## 2. Methods

All experiments were performed under a UK Home Office Licence and conducted according to the Animals Scientific Procedures Act 1986 (UK) and directive 2010/63/EU of the European Parliament guidelines on the protection of animals used for scientific purposes. All experiments were approved by the Institutional Animal Welfare and Ethical Review Body. Male wild-type (PKG1α-WT) or PKG1α Cys42Ser knock-in (C42S PKG1α KI) mice were bred and maintained inhouse.^13^ Male C57BL/6 mice (background for the PKG1α Cys42Ser mice) were purchased from Charles River Laboratories (Massachusetts, USA), and all mice were age- and body weight-matched.

### 2.1 Tissue harvest and blood sampling

C57BL/6 mice were administered a single i.p. injection of sodium nitrite (Sigma-Aldrich, Missouri, USA) dissolved in sterile saline (Baxter Healthcare, Deerfield, IL, USA; 0.9%) at various doses [0 (vehicle control), 0.1, 1.0, and 10 mg/kg body weight^9^^,^^10^]. Following nitrite treatment 1 or 24 h previously, mice were terminally anaesthetized [i.p. injection of pentobarbital sodium (300 mg/kg) mixed 50:50 with anticoagulant heparin (150 units)] and biological specimens were harvested. Blood was collected and supplemented with *N*-ethylmaleimide (NEM) and EDTA from a 10× stock in phosphate buffered saline to yield final concentrations of 10 mM NEM/1 mM EDTA and centrifuged for 10 min at 1000 *g* to separate plasma from blood cells. Plasma was then aliquoted, snap frozen in liquid nitrogen, and stored at −80°C until further analysis for nitrite/nitrate and cGMP. Thoracic aorta (conduit) and mesenteric resistance vessels were immediately isolated, dissected in ice-cold Krebs Henseleit buffer (KHB),^12^^,^^18^ divided into aliquots, snap frozen, and stored at −80°C until analysis for persulfides and H_2_O_2_. The liver was isolated *in situ*, perfused free of blood, snap frozen, and stored at −80°C for *in vitro* investigations of nitrite’s effects on catalase activity. Mice were euthanized by cervical dislocation.

### 2.2 Functional studies on isolated blood vessels

Conduit vessel rings were isolated from the thoracic aorta, and second-order mesenteric arteries (resistance vessels) from PKG1α WT and C42S PKG1α KI mice. Blood vessels were mounted in a tension myograph (Danish Myo Technology, Hinnerup, Denmark) containing KHB (37°C, pH 7.4) and gassed with 95% O_2_/5% CO_2_. After equilibration, vascular reactivity was normalized as previously described.^12^^,^^18^ Submaximal vasocontraction in both vessel types was induced by addition of phenylephrine (PE; Sigma-Aldrich, Missouri, USA) or U46619 (Cambridge Biosciences, Cambridge, UK), respectively, to the organ bath. Relaxation-response curves to sodium nitrite were constructed in the absence or presence of the selective soluble guanylate cyclase inhibitor, 1H-[1,2,4] oxadiazolo[4,3,-a]quinoxalin-1-one (ODQ; Sigma-Aldrich, Missouri, USA; 10 µM^19^) or the NO scavenger, 2-4-carboxyphenyl-4,4,5,5-tetramethylimidazoline-1-oxyl-3-oxide (CPTIO; Enzo Life Sciences, Exeter, UK; 1 mM^19^), and results expressed as a percentage of stable precontractile tone achieved.

### 2.3 Telemetric blood pressure monitoring *in vivo*

Mean arterial pressure (MAP) in conscious, freely moving PKG1α WT and C42S PKG1α KI mice was assessed using telemetry, as previously described.^13^ Briefly, mice were anaesthetized with 2% isoflurane in 0.5 l of oxygen per minute, and a TA11PA-C10 probe catheter (Data Science International, Minnesota, USA) was implanted in the aortic arch via the left aortic carotid artery. Pre- and post-operative analgesia (buprenorphine, subcutaneous injection of 0.1 mg/kg of body weight; Abbot Laboratories, Illinois, USA) was also provided, and the wellbeing of mice monitored daily. After 1 week recovery, mice were placed on the telemetric receivers, and MAP was recorded every 5 min. Blood pressures were measured throughout the day for 72 h before the start of the experiment. Mice then received a single i.p. injection of 0.1 mg/kg sodium nitrite dissolved in sterile saline and their blood pressure was monitored for further 24 h, followed by the administration of a successive 10-fold higher dose of nitrite at the same time on the subsequent 2 days while blood pressure monitoring continued.

### 2.4 Mass spectrometric analyses of tissue persulfide concentrations

High performance liquid chromatography-electrospray ionization tandem mass spectrometry (LC-ESI-MS/MS) analysis of tissue homogenates following HPE-IAM derivatization was used to determine CysSSH, GSSH, and inorganic persulfide (HSSH; disulfane), as previously described.^17^ Briefly, mesenteric and aortic tissues were homogenized with ice-cold 100% methanol solution containing 5 mM HPE-IAM and then incubated at 37°C for 30 min. The homogenates were then centrifuged at 15 000 *g* for 10 min to remove the protein pellet. After centrifugation, the aliquots of the supernatants were diluted with 0.1% formic acid containing known amounts of isotope-labelled internal standards. The samples were then analysed for CysSSH, GSSH, and HSSH by LC-ESI-MS/MS.

To assess the potential of H_2_O_2_ to induce the formation of persulfides from thiols and sulfide under various reaction conditions, l-cysteine (100 µM) or l-glutathione (100 µM), and sodium hydrosulfide (NaHS; 100 µM) was mixed with H_2_O_2_ (100 µM) in Tris/HCl (pH 7.5 or pH 9) or acetate buffer (pH 5.6) containing 2 mM HPE-IAM. The reaction solutions were incubated at room temperature for 10, 30, 60, 90, or 120 min and subsequently analysed for cysteine (CysSH), CysSSH, HSH, HSSH, GSH, and GSSH.

### 2.5 Plasma nitrite/nitrate and cyclic GMP determination

Nitrite and nitrate were quantified in plasma as previously described.^10^^,^^20^ Plasma cGMP was determined using a commercially available assay kit (KGE003 R&D systems, Minneapolis, UK).

### 2.6 Tissue hydrogen peroxide

Hydrogen peroxide concentrations were measured in tissue (mesenteric and thoracic aorta) using a commercially available assay kit (ab102500 Abcam, Cambridge, UK).

### 2.7 Tissue catalase activity

Tissue catalase activity was determined by monitoring the rate of H_2_O_2_ disappearance in the absence and presence of different nitrite concentrations (for order of reagent addition see Supplementary material online, *Figure S1D*). H_2_O_2_ concentrations were quantified using the horseradish peroxidase-mediated oxidation of phenol red and compared to a calibration curve with absorbance readings taken at 610 nm.^21^ For these experiments, frozen mouse liver aliquots were homogenized at 1:4 ratio (w/v) in EDTA-containing phosphate buffer pH 7.4 using a glass–glass Potter-Elvehjem tissue homogenizer immersed in an ice/water bath. The homogenization buffer was supplemented with NEM (10 mM, final concentrations) to minimize potential thiol-mediated metabolic conversion of nitrite to nitroso species. The homogenate was centrifuged (13 000 *g*, 15 min, 4°C), and kept on ice in the dark for up to 1 h for measurements of catalase activity, in the absence and presence of nitrite.

## 3. Results

### 3.1 Nitrite regulates mesenteric resistance vasorelaxation NO-independently during normoxia

**Figure 1:**
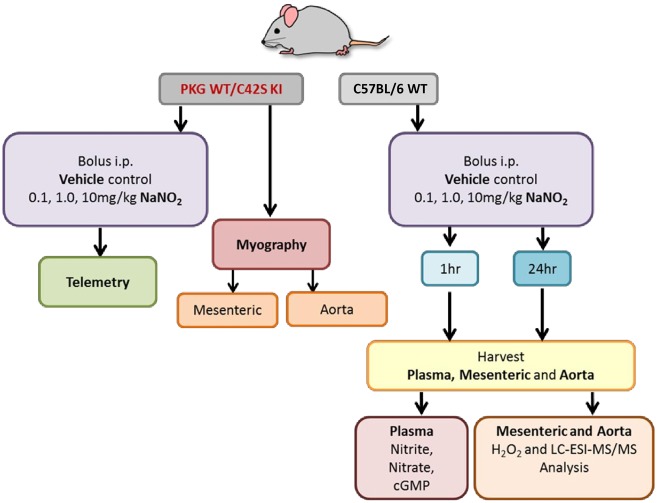
Schematic experimental design to assess nitrite-induced vasodilatory and blood pressure lowering effects as well as biochemical changes during normoxia.

**Figure 2:**
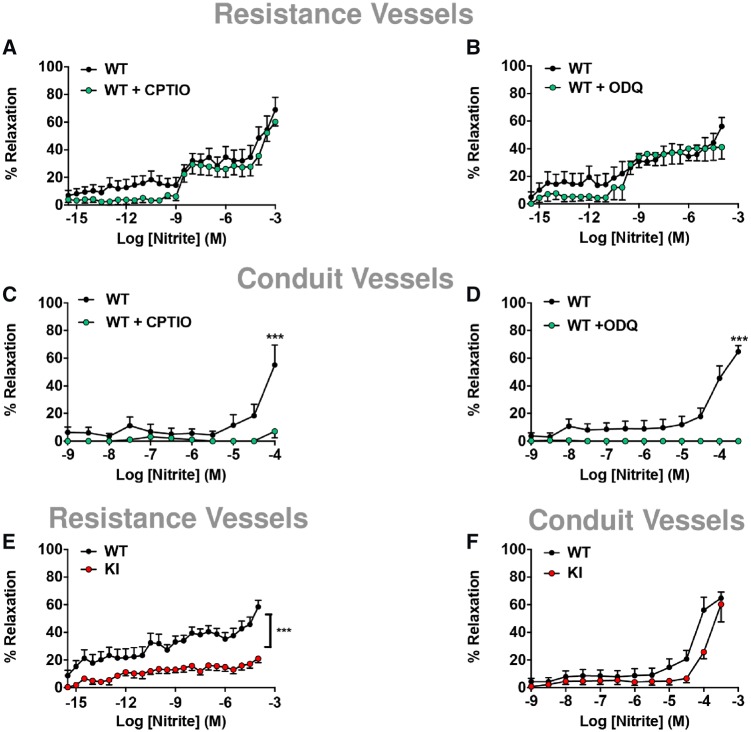
Mesenteric and thoracic blood vessels from PKG-1α WT and C42S PKG1α KI were mounted in myograph and sub-maximally constricted with U46619 and phenylephrine, respectively. Pre-incubation of isolated mesenteric resistance vessels from WT to (*A*) NO scavenger, carboxy-PTIO (CPTIO; 1 mM; *n* = 15) or (*B*) soluble guanylate cyclase inhibitor (sGC), ODQ (10 µM; *n* = 14) did not attenuate the vasodilatory response to nitrite in WT mice. Concentration response curves to nitrite in isolated thoracic aorta. Vasorelaxant effects of nitrite was (*C*) NO-dependent (*n* = 7) and (*D*) sGC-dependent in WT mice (*n* = 11). Concentration response curves to sodium nitrite in (*E*) isolated mesenteric resistance vessels from both genotypes (*n* = 19). Nitrite was significantly less effective as a vasodilator in C42S PKG1α KI mice, but not in the (*F*) thoracic aorta (conduit vessels; *n* = 13–14). Data are expressed as mean ± S.E.M. ***P ≤ 0.001 as determined by two-way ANOVA with Bonferroni’s test (*A–F*).

To determine whether in the presence of oxygen nitrite mediates vasorelaxation in an NO-independent or independent fashion, we first constructed cumulative concentration-response curves to nitrite in isolated pre-constricted mesenteric resistance vessels mounted in wire-myographs (*Figure 1*). Pre-incubation of resistance vessels from the wild-type (WT) mice with either the NO-scavenger, carboxy-PTIO or the sGC inhibitor, ODQ failed to abrogate vasorelaxation in the resistance vessels from WT mice (*Figure 2A *and* B*), indicating that the effects of nitrite in this tissue were largely NO/sGC independent. In contrast, when wire-myography was performed in conduit vessels (thoracic aorta) nitrite’s vasorelaxant effects were clearly NO/sGC-dependent (*Figure 2C *and* D*).

In light of the above, we hypothesized that nitrite-mediated vasorelaxation may perhaps occur via PKG1α oxidation, independent of the classical NO-cGMP pathway. To explore this possibility, we used a ‘redox-dead’ genetic variant of PKG1α whereby the important regulatory SH moiety has been mutated to a redox inactive OH group (by replacing a single cysteine, Cys42, against serine).^13^ Using this C42S PKG1α knock-in (KI) mouse model and WT littermates as a comparator, we next investigated the molecular target involved in nitrite-mediated vasorelaxation (*Figure 1*). Nitrite-relaxed WT vessels in a concentration-dependent fashion, but did not relax resistance vessels from C42S PGK1α KI mice (*Figure 2E*); whereas in the conduit vessels, nitrite was not mediated through PKG1α oxidation (*Figure 2F*).

**Figure 3:**
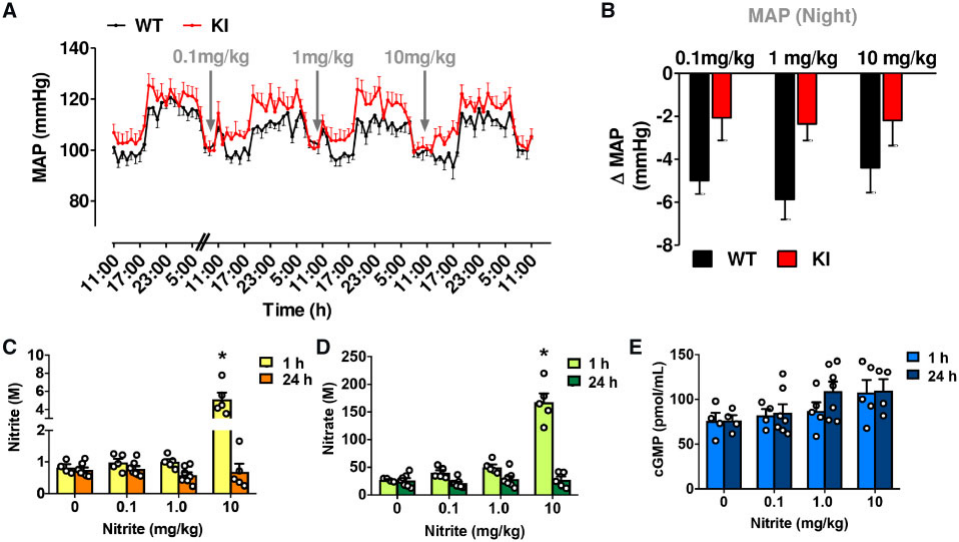
C42S PKG1α KI mice are resistant to nitrite-induced blood pressure lowering compared with age-matched WT littermates, as measured by *in vivo* telemetry (*n* = 6 WT and 7 KI). (*A*) Comparison of the 24 h averaged changes in MAP in both genotypes following bolus administration of sodium nitrite (0.1, 1, and 10 mg/kg). WT mice, but not for KI mice, there was a significant difference in blood pressure lowering effect with each nitrite dose when compared to baseline. (*B*) Delta indicates the difference between baseline and response to treatment at night. In separate group of WT mice (C57BL/6), following bolus i.p. administration with sodium nitrite (vehicle control, 0.1, 1, and 10 mg/kg) for 1 or 24 h treatment, plasma samples were taken (*C*) nitrite, (*D*) nitrate, and (*E*) cGMP (*n* = 4–7 mice/group). Data are represented as mean ± S.E.M. *P ≤ 0.05 when WT mice compared to each nitrite dose with baseline (repeated measures one-way ANOVA, *A*). *P ≤ 0.05 between two different genotypes was determined by repeated measures two-way ANOVA (*B*). Significant time and concentration interaction for plasma nitrite and nitrate, as determined by two-way ANOVA. Following Tukey’s multiple comparisons test 10 mg/kg sodium nitrite was significant when compared to control vehicle, 0.1 and 1 mg/kg (*P ≤ 0.05; *C* and *D*). There was no significant difference between nitrite concentration for cGMP levels at either time (*P* > 0.05; two-way ANOVA followed by Tukey’s multiple comparisons test; *E*).

To translate our findings into the *in vivo* setting, we administered i.p. injections of sodium nitrite (0.1, 1, and 10 mg/kg) in WT mice on three consecutive days following 72 h continuous telemetric arterial blood pressure monitoring. Compared to the hourly averages for the 3 days baseline blood pressure measurements shown, reductions in blood pressure were not apparent until 4–5 h after the nitrite application (*Figures 1*and*3A*; WT-black trace), but blood pressure remained reduced for almost 24 h, especially during the nocturnal period when the animals were most active (*Figure 3B*, WT-black bars). Unexpectedly, the magnitude of blood pressure lowering by the three doses was similar.

We next assessed the *in vivo* blood pressure lowering effects of nitrite in redox-dead C42S PKG1α KI using the same treatment regime as in their PKG-WT littermates (see above). Of note, the C42S PKG1α KI had modestly, but consistently higher mean blood pressure than the WT.^13^ In contrast to the long-lasting hypotensive effects seen with nitrite in WT mice, C42S PKG1α KI animals did not show a blood pressure lowering effect. *Figure 3A* compares the 24 h MAP data in both genotypes following i.p. administration of sodium nitrite (0.1, 1, and 10 mg/kg). *Figure 3B* is further simplified to illustrate the delta difference between baseline and response to nitrite treatment at night. There was a significant difference between the two genotypes (*P < 0.05 determined by repeated measures two-way ANOVA). These results are consistent with the notion that the ability of nitrite to elicit persistent hypotension depends on its capacity to oxidatively activate PKG1α.

In a separate group of C57BL/6 mice, the same nitrite injection regime was used and blood samples were taken 1 and 24 h post-treatment for measurement of plasma nitrite, nitrate, and cGMP (*Figures 1*and*3C–E*). Plasma nitrite and nitrate acutely increased in a dose-dependent manner, and this was statistically significant at the highest dose (i.e. 3–4 h before a BP-lowering effect was observed), as determined by two-way ANOVA. Following Tukey’s multiple comparisons test 10 mg/kg sodium nitrite, plasma nitrite was significantly higher at 1 h when compared with control vehicle, 0.1 and 1 mg/kg; *P < 0.05). However 24 h later plasma nitrite, nitrate, and cGMP were similar to baseline concentrations even at the highest dose (despite persistent BP reduction persisting for almost 24 h); these changes were consistent with our previous results in rats subjected to i.p. nitrite administration, demonstrating that plasma nitrite levels rapidly increased, but returned close to baseline values in ∼1 h.^9^^,^^10^

### 3.2 Nitrite increases hydrogen peroxide levels

**Figure 4:**
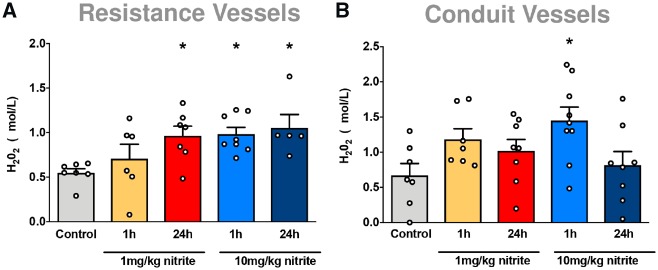
In separate group of mice (C57BL/6), mesenteric resistance and thoracic aorta blood vessels were harvested following bolus i.p. administration of vehicle control or sodium nitrite (1, or 10 mg/kg) in WT mice (C57BL/6) for 1 or 24 h. Hydrogen peroxide (H_2_O_2_) levels were measured in (*A*) mesenteric resistance vessels (*n* = 5–8) and (*B*) thoracic aorta (*n* = 7–9). Data are expressed as mean ± S.E.M. *P ≤ 0.05 as determined by one-way ANOVA with Dunnett’s test.

We next focused on the mechanism of nitrite-mediated vasorelaxation of resistance arteries and blood pressure lowering. Nitrite has the potential to inhibit catalase,^22^^,^^23^ which may conceivably lead to an increased oxidative tone by reducing the breakdown of endogenously produced H_2_O_2_. This action was confirmed in complementary studies in which nitrite was found to be capable of inhibiting catalase activity in hepatic tissue *in vitro* (Supplementary material online, *Figure S1*). In order to determine whether this can also occur in vascular tissue, we measured *ex vivo* H_2_O_2_ concentrations in blood vessels harvested 1 and 24 h after nitrite pre-treatment *in vivo* (*Figure 1*). As depicted in *Figure 4A*, nitrite caused a significant and similar increase in steady-state concentrations of H_2_O_2_ in mesenteric resistance vessels with both 1 and 10 mg/kg nitrite at 1 h, which persisted at 24 h. Whilst nitrite also triggered an increase of this oxidant in the aorta at 1 h, H_2_O_2_ concentrations had returned to normal at 24 h (*Figure 4B*), revealing kinetic differences in H_2_O_2_ handling between these two types of vessels.

### 3.3 Nitrite increases CysSSHs and GSSH in resistance vessels during normoxia

**Figure 5:**
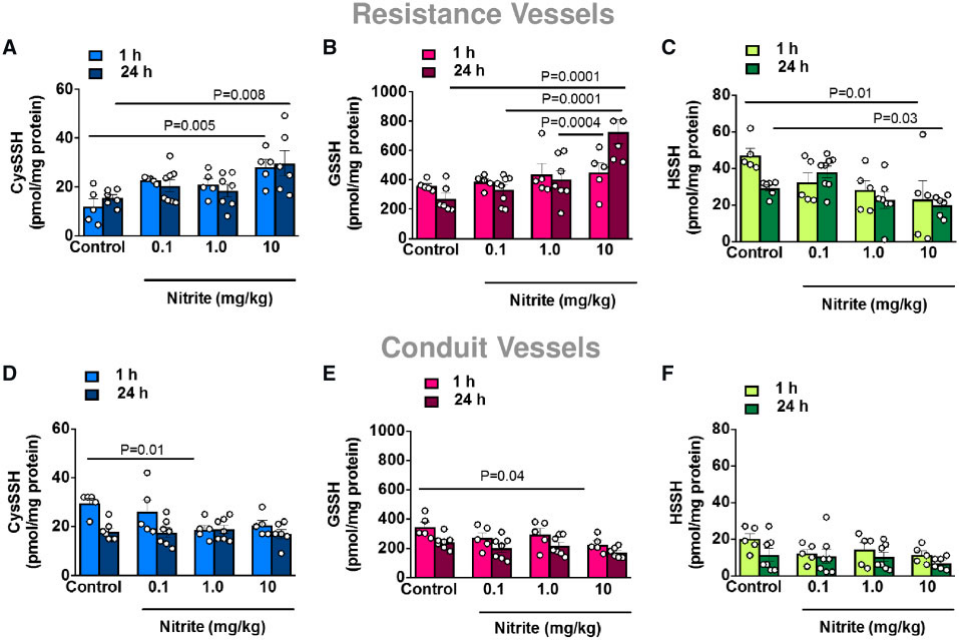
Mesenteric resistance and thoracic aorta blood vessels were harvested from WT mice (C57BL/6) treated with bolus i.p. administration of vehicle control or sodium nitrite (0.1, 1, and 10 mg/kg) for 1 or 24 h. The levels of persulfides (CysSSH, GSSH, and HSSH) were analysed by liquid chromatography-electrospray ionization tandem mass spectrometry (LC-ESI-MS/MS) following derivation with β-(4-hydroxyphenyl)ethyliodoacetamide (HPE-IAM). Data are represented as mean ± S.E.M (*n* = 5–8). *P ≤ 0.05 as determined by two-way ANOVA with Tukey’s test (*A–F*).

**Figure 6:**
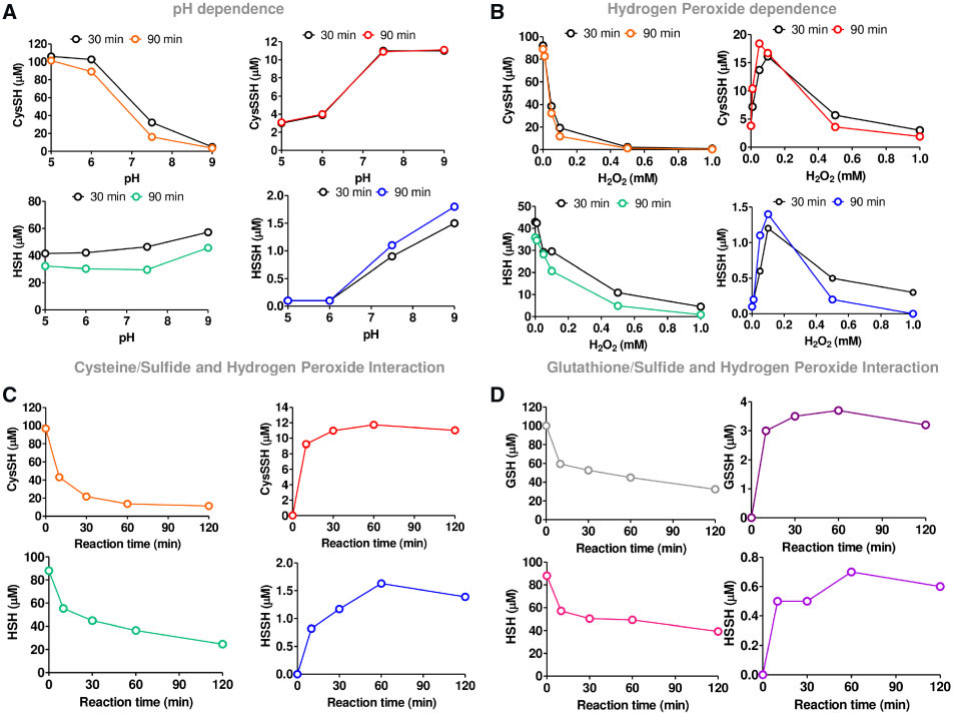
Cysteine persulfide (CysSSH) and polysulfides formation during various reactions. (*A*) pH conditions (5, 6, 7.5, and 9) in the presence of cysteine (100 µM), sulfide (NaHS; 100 µM), hydrogen peroxide (H_2_O_2_; 100 µM) in 50 mM Acetate buffer (pH 5 and 6), or Tris/HCL (pH 7.5 and 9) or HPE-IAM (2 mM). (*B*) H_2_O_2_ concentrations (0, 0.01, 0.05, 0.1, and 1 mM) in the presence of cysteine (100 µM), NaHS (100 µM) in 50 mM Tris/HCL pH 7.5. Similar results were obtained with glutathione/sulfide mixtures, although final concentrations of GSSH achieved were less compared to the corresponding cysteine analogue. All reactions were also accompanied by the formation of thiosulfate (S_2_$O_{3}^{2\text{−}}$) and small concentrations of the corresponding trisulfide (data not shown). (*C*) Interaction of cysteine (100 µM) or (*D*) Glutathione (GSH; 100 µM) in the presence of NaHS (100 µM), H_2_O_2_ (100 µM) in 50 mM Tris/HCL (pH 7.5). Data presented are from one single independent experiment for each condition.

The results presented thus far indicate that nitrite produces H_2_O_2_ and induces vasorelaxation by oxidation of PKG1α. We next examined whether there was a possible intermediate that links these effects. We surmised that nitrite may have the ability to oxidatively activate PKG1α via the generation of polysulfur species. To establish whether nitrite generates polysulfur species *in vivo*, mesenteric resistance and thoracic aorta blood vessels were harvested from WT mice (C57BL/6) treated with bolus i.p. administration of vehicle control or sodium nitrite (0.1, 1, and 10 mg/kg) for 1 or 24 h. The levels of persulfides (CysSSH, GSSH, and HSSH) were analysed by LC-ESI-MS/MS following derivation with β-(4-hydroxyphenyl)ethyliodoacetamide (HPE-IAM). We observed dose-dependent increases in CysSSH and GSSH in the resistance vessels (*Figure 5A *and* B*) with progressively lower concentrations of HSSH (*Figure 5C*). In contrast, we observed no changes in CysSSH, GSSH, or HSSH formation in the conduit vessels (*Figure 5D–F*, respectively). In further *in vitro* studies, we sought to confirm experimentally that H_2_O_2_ is capable of inducing polysulfidation when inorganic sulfide and cysteine are present. This was indeed found to be the case under a variety of pH and H_2_O_2_ conditions (*Figures 6A–C*), and similar results were observed with glutathione/sulfide/H_2_O_2_ (*Figure 6D*).

## 4. Discussion

We have previously shown that the conversion of nitrite to NO is inhibited by oxygen,^8^ and the mechanism(s) through which it exerts its pharmacological effects under normoxia remain(s) poorly understood. Whilst blood pressure lowering effects of nitrite have been shown in various animal models and clinical studies, to the best of our knowledge, the impact of long-lasting effects of single bolus application of nitrite during physiological conditions has not been investigated. In the present study, we demonstrate that a single bolus application of nitrite elicits a long-lasting blood pressure lowering effect that is unrelated to the generation of NO. We furthermore show that a marked temporal dissociation exists between plasma nitrite and cGMP concentrations, and the blood pressure lowering effects of i.p. injections of nitrite, with the hypotensive effects being delayed in onset vs. circulating nitrite/cGMP concentrations and persisting despite the return of the latter to baseline values. These observations differ considerably from the short-lived BP-lowering effects of a single intravenous injection of nitrite, thus supporting the role of a non-canonical vasodilator effect via a mechanism that is independent of the NO-cGMP pathway. Our results are consistent with the notion that nitrite has signalling properties in its own right^9^ and provides evidence for the notion that nitrite can act as an indirect oxidant. Our next efforts focused on how nitrite might induce vasorelaxation and lower blood pressure. Using a combination of biochemical analytical tools and a genetic KI mouse model, we finally demonstrate that the blood pressure lowering effects of nitrite are mediated by PKG1α oxidation following H_2_O_2_ and cysteine/glutathione persulfide formation in the resistance vasculature. A conceivable explanation for which evidence exists in the literature is that nitrite acts as an indirect oxidant that mediates oxidation of other biological targets.

We previously demonstrated that oxidants such as H_2_O_2_ can activate PKG1α independently of the classical NO-sGC-cGMP pathway by forming a disulfide bond at Cys42 to dilate resistance vessels and lower blood pressure.^13^ Consistent with this finding, PKG1α-KI mice in which WT kinase has been replaced by a ‘redox-dead’ serine residue (C42S mutant) cannot transduce oxidant signals because their PKG does not harbour the redox-sensing thiol, rendering these mice unable to lower blood pressure via an oxidative mechanism.^13^ Independent of this, we demonstrated that nitrite chemically/functionally interacts with H_2_S^24^ and that a single bolus application of nitrite triggers long-lasting alterations in myocardial redox status with dramatic alterations in the cardiac mitochondrial proteome.^10^ We here sought to integrate these different strands of information and assessed whether nitrite could produce a metabolic signature consistent with a process involving thiol/sulfide oxidation and whether this might be associated with PKG1α dimerization to mediate vasorelaxation and blood pressure reduction.

Whilst the resistance vessels are the primary determinant of diastolic and mean blood pressure, most vascular studies investigated the effects of nitrite in the aorta and have shown NO-dependent effects. Here, we assessed both the aorta and the mesenteric resistance vessels from WT and C42S PKG1α KI mice. Unlike the aorta, it was evident in the isolated resistance vessels from WT mice that nitrite-mediated vasodilatory effects were NO-sGC independent. Moreover, the mesenteries from mice expressing the ‘redox-dead’ C42S form of PKG1α were relatively insensitive in their vasodilatory response to nitrite compared to WT. Based on these findings, we anticipated that the long-lasting *in vivo* blood pressure lowering effect of nitrite would also be diminished in the KI mice. Indeed, this was found to be the case because nitrite decreased blood pressure via non-canonical oxidative activation of PKG1α, whereas the KI mice were resistant to this hypotensive response.

Nitrite has the ability to reduce the rate of H_2_O_2_ decomposition by binding to the haem moiety of catalase thereby inhibiting enzyme activity^23^^,^^25^; thus we reasoned that nitrite may inhibit catalase activity and possibly increase H_2_O_2_ levels, especially in resistance vessels, and this may be responsible for the vasoactivity observed. Exploratory studies on the inhibition of catalase activity by nitrite in mouse liver tissue *in vitro* confirmed that this may be a viable mechanism*.* In the vasculature, H_2_O_2_ concentrations were found to remain elevated for prolonged periods of time in the mesenteric resistance vessels when compared with conduit vessels. Although these observations would seem to provide a mechanism that could explain the NO-independent actions of nitrite as a vasorelaxant, previous studies reporting H_2_O_2_-mediated PKG1α activation required high concentrations of this oxidant,^12^ suggesting the effect may not be entirely due to H_2_O_2_ directly.

Reactive sulfur-containing species, such as CysSSH have been known to exist endogenously, but the physiological relevance of these species remains incompletely understood. CysSSH evidently generates GSSH, and previous work from our group has shown that these reactive species act as potent scavengers of oxidants such as H_2_O_2_, which contributes to cellular redox signalling.^16^ Furthermore, Stubbert *et al.*^14^ reported that polysulfur species derived from H_2_S oxidation are capable of activating PKG1α via oxidation. Therefore, the generation of polysulfur species may represent an NO-independent mechanism of PKG1α activation, which could mediate vasorelaxation. Thus, the ability for nitrite to oxidatively activate PKG1α may be due to additional generation of polysulfur species. Indeed, there is precedence for the interaction of nitrite with sulfide in forming another reactive sulfur species, nitrosopersulfide, in the literature.^24^ Previous reports indicate that analysis of CysSSH, GSSH, and HSSH (disulfane) can serve as detectable surrogates for overall levels of cellular polysulfur species, since they are all in dynamic equilibrium.^26^^,^^27^ Thus, to test the hypothesis that nitrite generates polysulfur species *in vivo*, the concentrations of all three compounds were determined by mass spectrometry in conduit and mesenteric resistance vessels of WT mice 1 and 24 h after nitrite administration. We observed dose-dependent increases in CysSSH and GSSH in the resistance vessels with progressively lower concentrations of HSSH. Since disulfane is highly reactive, it may immediately react as intracellular disulfide concentrations rise to produce CysSSH and GSSH. In contrast to our findings in resistance vessels, no increases in CysSSH or GSSH were apparent in conduit vessels. These observations demonstrate that bolus applications of nitrite trigger polysulfur species formation through an NO-independent mechanism already at low-to-moderate pharmacological doses with marked differences between vascular beds. Interestingly, persulfide formation from the resistance vessels matched the temporal profile of nitrite effects on H_2_O_2_ and blood pressure.

Thus far, we demonstrated that nitrite is capable of eliciting increases in H_2_O_2_ and polysulfur species, with greater propensity for resistance vessels than conduit vessels. It is intriguing to speculate that both events are related and that the source of the polysulfur species is via H_2_O_2_-mediated oxidation of reduced thiol species. In further *in vitro* studies, we thus sought to confirm experimentally that H_2_O_2_ is capable of inducing polysulfidation when inorganic sulfide and cysteine are present. This was indeed found to be the case under a variety of conditions (pH dependence and H_2_O_2_ concentrations) and similar results were observed with glutathione/sulfide/H_2_O_2_. Taken together, these reactions strongly suggest that nitrite can indirectly contribute to polysulfide formation by acting as an amplifier of endogenous H_2_O_2_ concentrations.

Although it is intriguing to speculate that nitrite may elicit vasorelaxation via its capacity to raise H_2_O_2_ levels which subsequently give rise to organic persulfides which, in turn, activate PKG1α, it is important to reiterate that detection of CysSSH/GSSH is merely indicative of the general presence of polysulfur species (including protein per- and polysulfides)^26^ and that these particular species are not necessarily solely responsible for the biological effects observed herein. What detection of these species does indicate, however, is the presence of an oxidizing environment consistent with the notion of oxidative PKG1α activation. Due to the analytical challenges and rapid interconversion of reactive sulfur species it is currently not possible to disentangle these reaction channels further. Nevertheless, the key findings of this work are that nitrite—when applied i.p.—is capable of creating an oxidizing environment *in vivo*, as evidenced by increases in H_2_O_2_ and polysulfide concentrations in vascular tissue, favouring PKG1α dimerization, and that these effects occur primarily in resistance vessels. While it has been reported that nitrite is capable of inhibiting catalase (which we confirm to also occur in tissues), and this is a possible pathway through which nitrite creates an oxidizing environment, the exact mechanism(s) by which this occurs are not established. We cannot exclude, for example, that nitrite also increases vascular NADPH oxidase activity^28^ and/or up-regulates SOD^29^ and/or increases superoxide/H_2_O_2_ formation by interfering with electron transfer processes in the mitochondria; these possibilities warrant further investigation. Furthermore, increases in resistance vessel H_2_O_2_ concentration were similar at 1 and 10 mg nitrite, which may explain the absence of dose-response for blood pressure lowering over the range of doses explored.

**Figure 7:**
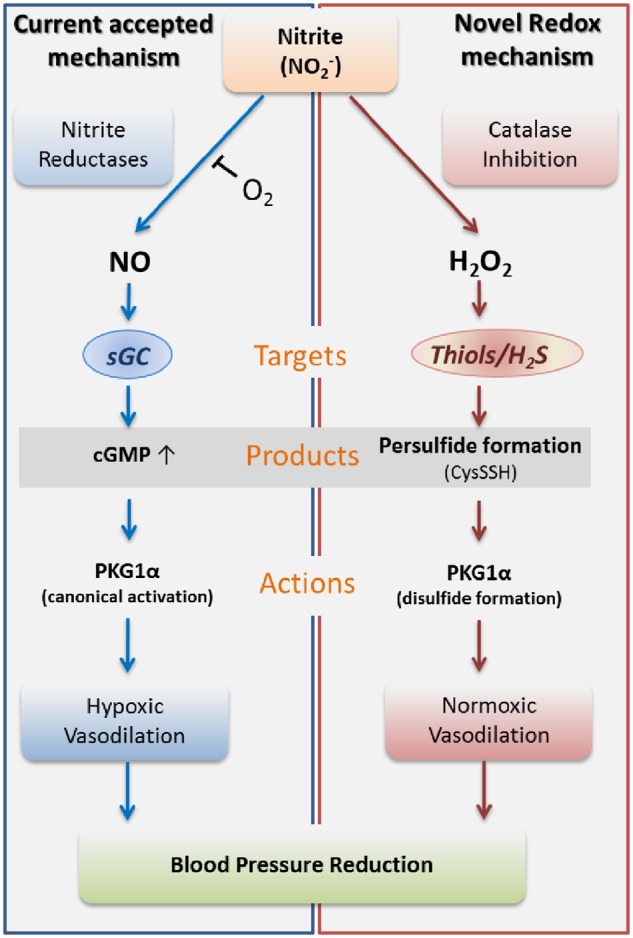
Proposed mechanism of action involved in nitrite-mediated vasorelaxation, including the canonical nitrite reduction pathway (left—blue panel) and the novel nitrite oxidation pathway (right—red panel).

The ability of nitrite to elicit its vasoactive effects to a greater extent in resistance vs. conduit vessels is intriguing and requires independent confirmation. This mechanism is at odds with nitrite’s currently accepted mode of action (*Figure 7*) and represents an unusual case of non-canonical pharmacology whereby a short-lived pro-oxidant elicits long-lasting hemodynamic effects *in vivo.* To our knowledge, this is the first report for such a mechanism in resistance vessels *in vivo*.

These findings may potentially be relevant not only to pharmacological, but also to physiological concentrations to nitrite. A previous study reported that antibacterial mouthwash reduced plasma nitrite and increased blood pressure in treated hypertensive patients.^30^ Herein, we demonstrated that the delayed but sustained blood pressure lowering effect of nitrite was markedly attenuated in the C42S PKG1α KI vs. the WT mice. Furthermore, we also confirm a previous report that the baseline blood pressure was higher in the C42S PKG1α KI mice.^13^ This could potentially be related to effects of physiological concentrations of nitrite on PKG oxidation state or may be consistent with a previous report of impaired acetylcholine mediator effects observed in the C42S PKG1α KI.^13^ Mesenteries from KI mice were deficient in their responses to H_2_O_2_ or acetylcholine, as well as the latter in the presence of L-NAME and indomethacin to remove vasodilation by classical nitric oxide- or prostacyclin-dependent mechanisms.^6^ This deficiency in acetylcholine-dependent relaxation in the C42S PKG1α KI is consistent with the endothelium-derived hyperpolarizing factor-dependent component of vasodilation induced by this neurotransmitter being significantly mediated by H_2_O_2_ generation.^31^ Indeed, evidence was presented that C42S PKG1α is deficient in activation by cGMP, although whether this mutation impacts the affinity with cGMP was not determined, and this was suggested to underlie the loss of function observed in C42S PKG1α KI mice.^32^ However, the studies comparing the response of isolated resistance vessels of these mutant mice to WT controls, showed their vasodilation to NO donors or a cell-permeable cGMP analogue were the same.^13^

Although oxidation of C42 has been implicated in the targeting and the oxidation of PKG1α, there is additional complexity because of the potential roles of other redox-active cysteines and the interrelationship between cGMP-binding and kinase oxidation. It may be that C42 is more important for targeting the kinase to its substrates, and that cGMP or oxidation of another cysteines is required for kinase competency.^33^ For example, nitroxyl donors were shown to induce a disulfide at C42 as well as an intra-disulfide between C117 and 195 in the high-affinity cGMP-binding site of PKG1α—with the latter oxidation mediating most of the activation induced by this nitro-oxidative intervention.^34^ C195 was also shown to adduct the electrophile 8-nitro-cGMP, with this S-guanylation modification leading to persistent activation of the kinase.^35^ Hyperoxidation of C117 has been implicated as a mechanism of PKG1α activation by H_2_O_2_,^36^ whilst activation by this oxidant was not observed by others.^32^ In summary, the redox control of PKG1α is complex, with our understanding continuing to evolve as contemporary studies on the subject emerge. Nevertheless, a casual role for PKG1α C42 oxidation in oxidant or endothelium-derived hyperpolarizing factor-dependent vasodilation is supported by a number of studies.

The generation of polysulfur species is dependent on the presence of cysteine and/or H_2_S.^17^^,^^37^ Thus, if PKG1α activation is polysulfide-mediated (as our *in vivo* experiments suggest) the generation of H_2_S in the target tissues becomes an important factor for the action of nitrite and may contribute to the tissue specificity of the observed effects. The overall outcome of these reactions on the effective concentrations of CysSSH and related species (and thus the net effect on physiological function) appears to be complex and tissue/organ-specific, as illustrated by the differences in regulation between resistance and conduit vessels in the present study. The factors contributing to these differences (e.g. oxygen tension, oxidative stress) warrant further investigation. 

In summary, this study reports a novel finding, whereby under physiological conditions nitrite induces long-lasting blood pressure lowering effects independent of NO-induced cGMP elevations. We show that nitrite lowers blood pressure by mediating oxidation of PKG1α. This mechanism has been shown by using ‘redox-dead’ C42S PKG1α KI mice, which cannot activate oxidant signals. We observed dose-dependent vasorelaxant responses to nitrite in the PKG1α WT isolated resistance vessels, but the vasodilatory response to nitrite were blunted in the C42S PKG1α KI mice. These findings were further substantiated by monitoring blood pressure *in vivo*, since nitrite was unable to lower blood pressure in the redox-dead C42S PKG1α KI mice compared with WT littermates. We also identified that the effect of nitrite was mediated via the generation of H_2_O_2_, with consecutive production of persulfide species (CysSSH and GSSH) in the resistance vessels, which oxidatively activate PKG1α. Our study concludes that a single bolus application of nitrite has the ability to act as a redox regulator during physiological conditions to induce long-lasting blood pressure lowering effects, providing a new prospect for therapeutic strategy in treating the global healthcare burden of hypertension. Future investigations are warranted to address the significance of this redox pathway in hypertensive and other cardiovascular disease models. Furthermore, our findings raise the possibility of identifying other molecules capable of modulating this novel pathway that may have therapeutic potential as hypertensive agents.

## Authors’ contributions

M.Ma., M.F., and M.P.F designed the studies. M.Ma., P.E, O.P., J.J.G, N.D.G, A.B, R.M.H, K.G. designed, carried out, and analysed the PKG1α and C57BL/6 *in vivo* experiments. M.C.K. contributed pilot data and provided technical guidance for mice telemetry experiments. O.P. carried out and analysed PKG1α telemetry experiments. M.Ma., M.F., and K.G. designed, carried out, and analysed the H_2_O_2_ experiments. B.O.F. and M.Mi. contributed to nitrite, nitrate, and cGMP assays and performed exploratory catalase inhibition/polysulfide formation experiments. M.Ma., M.F., T.A., and T.I. designed and carried out *in vitro* polysulfide experiments and analysed *in vivo* tissue samples. All authors interpretated the data. M.Ma., M.F., and M.P.F. wrote the original manuscript and all authors reviewed the revised manuscript.

## Supplementary Material

cvz202_Supplementary_Materials

## Notes

**Time for primary review: 1 day**

## Notes

Translational perspective Despite current pharmacotherapies and interventional procedures, arterial hypertension remains a global health burden. Thus, novel therapeutic interventions are urgently required. Nitrite exerts vasorelaxant effects of potential therapeutic relevance under normal physiological conditions, yet the mechanism of action remain unknown. Here, we present evidence that in normoxia nitrite lowers blood pressure via a novel redox mechanism. This occurs independent of nitric oxide by generating hydrogen peroxide and persulfide, which subsequently activates PKG1α by oxidation. Targeting this novel pathway may provide new prospects for anti-hypertensive therapy and other cardiovascular-related diseases.
