# Supplementary material for: Long-lasting blood pressure lowering effects of nitrite are NO-independent and mediated by hydrogen peroxide, persulfides, and oxidation of protein kinase G1α redox signalling
Source: Cardiovasc Res. 2019 Aug 1;116(1):51–62. doi: 10.1093/cvr/cvz202 (PMC6918062; doi:10.1093/cvr/cvz202)
Supplement: cvz202_Supplementary_Materials [file cvz202_supplementary_materials.zip › cvz202-suppl_data/cvz202_Supplementary_Material.docx]

**Long-lasting blood pressure lowering effects of nitrite are NO-independent and mediated by hydrogen peroxide, persulfides and oxidation of protein kinase G 1α redox signaling**

Martin Feelisch^1^, Takaaki Akaike^2^, Kayleigh Griffiths^6^, Tomoaki Ida^2^, Oleksandra Prysyahna^3^, Joanna J Goodwin^6^, Nicholas D Gollop^4,6^, Bernadette O Fernandez^1^, Magdalena Minnion^1^, Miriam M Cortese-Krott^5^, Alessandra Borgognone^6^, Rosie M Hayes^6^, Philip Eaton^4^, Michael P Frenneaux^4^, Melanie Madhani^6^*

^1^Clinical and Experimental Sciences, Faculty of Medicine, University of Southampton, Southampton, UK. ^2^Department of Environmental Medicine and Molecular Toxicology, Tohoku University Graduate School of Medicine, Sendai, Japan. ^3^Department of Cardiology, Cardiovascular Division, King’s College of London, London, UK.  ^4^Norwich Medical School, University of East Anglia, Norwich, UK.  ^5^ Division of Cardiology, Heinrich Heine University, Düsseldorf, Germany. ^6^Institute of Cardiovascular Sciences, University of Birmingham, Birmingham, UK.

These authors contributed equally: Martin Feelisch, Takaaki Alaike, Philip Eaton, Michael Frenneaux and Melanie Madhani

**Short title:** Nitrite-induced hypotension occurs via H_2_O_2_ and cysteine persulfide formation and PKG1α oxidation

**Word count:** 7667 (incl abstract, main text, figure legends & references)

*correspondence to:

Melanie Madhani, PhD

Institute of Cardiovascular Sciences

The Medical School

University of Birmingham

Birmingham, UK

E-mail: m.madhani@bham.ac.uk

**Supplementary Figure 1.** Inhibition of catalase activity by nitrite in mouse tissue (C57BL/6) in *vitro*. **A**. Concentration dependence of nitrite-induced catalase inhibition in murine liver homogenate (1:4; w:v); inset showing the same data plotted in a semi-logarythmic fashion. **B**. Effects of 1 mM nitrite on liver tissue catalase activity by monitoring remaining H_2_O_2_ (starting conc. 1, 5 or 10 mM) 30 and 90s following addition of different peroxide concentrations to 1:4 liver homogenate. **C**. Near complete inhibition of catalase activity by 1 mM nitrite in diluted liver homogenate (1:500; w:v; 50 µM H_2_O_2_ starting conc). Data represent means ± SD from representative experiments repeated at least twice with qualitatively identical outcome. **D**. Sequence of reagent additions for measurements in neat and diluted liver homogenate
