# Supplementary figures and images for: Long-lasting blood pressure lowering effects of nitrite are NO-independent and mediated by hydrogen peroxide, persulfides, and oxidation of protein kinase G1α redox signalling
Source: Cardiovasc Res. 2019 Aug 1;116(1):51–62. doi: 10.1093/cvr/cvz202 (PMC6918062; doi:10.1093/cvr/cvz202)

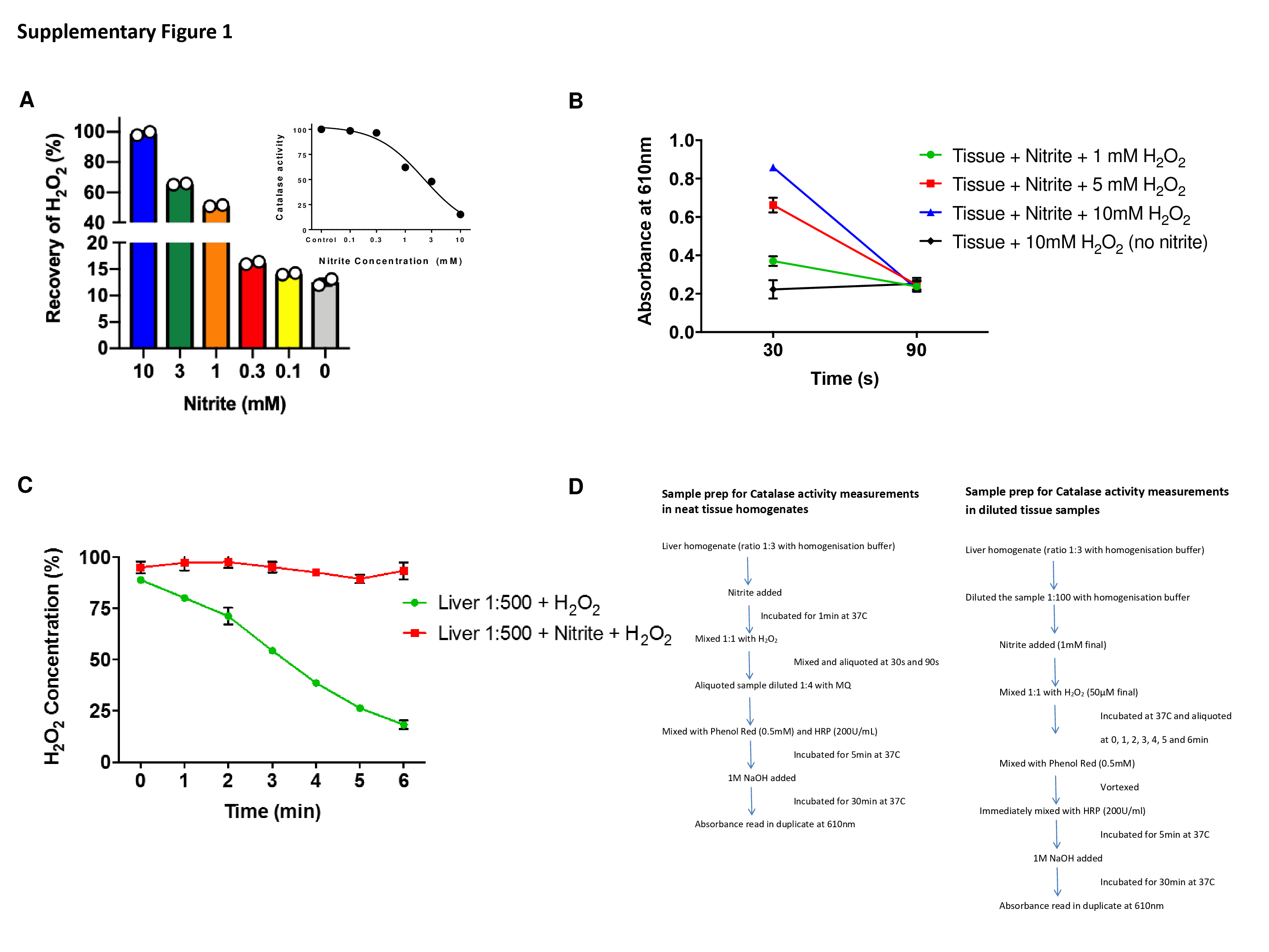

Supplement: cvz202_Supplementary_Materials [file cvz202_supplementary_materials.zip › cvz202-suppl_data/cvz202_Supplementary_Figure_1.tiff]
